# The effect of autologous platelet rich plasma on tenocytes of the human rotator cuff

**DOI:** 10.1186/s12891-018-2339-5

**Published:** 2018-11-30

**Authors:** Stephan Pauly, Franka Klatte-Schulz, Katharina Stahnke, Markus Scheibel, Britt Wildemann

**Affiliations:** 1 Julius Wolff Institut, Center for Musculoskeletal Surgery, Charité-Universitaetsmedizin, Augustenburger Platz 1, 13353 Berlin, Germany; 2 Berlin-Brandenburg Center for Regenerative Therapies, Charité-Universitaetsmedizin, Berlin, Germany; 3 Department of Experimental Traumatology, Universitaetsklinikum Jena, Jena, Germany

**Keywords:** PRP, Platelet rich, Rotator cuff, Tenocyte, Tendon cell, Autologous, Growth factor

## Abstract

**Background:**

Platelet rich plasma (PRP) is widely used in rotator cuff repairs but its effect on the healing process is unclear. Several cell culture studies on the effect of allogenic PRP have reported promising results but are not transferable to clinical practice.

The aim of the present study is to assess the possible effect of autologous PRP on rotator cuff tendon cells. The amount of growth factors involved with tendon-bone healing (PDGF-AB, IGF-1, TGF-β1, BMP-7 and -12) is quantified.

**Methods:**

Rotator cuff tissue samples were obtained from (*n* = 24) patients grouped by age (>/< 65 years) and sex into four groups and cells were isolated and characterized. Later, autologous PRP preparations were obtained and the effect was analyzed by means of cell proliferation, collagen I synthesis and expression of collagen I and III. Furthermore, the PRPs were quantified for growth factor content by means of platelet-derived growth factor (PDGF-AB), insulin-like growth factor (IGF-1), transforming growth factor (TGF-β1), as well as bone morphogenetic protein (BMP) -7 and − 12.

**Results:**

Cell proliferation and absolute synthesis of collagen I were positively affected by PRP exposure compared to controls (*p* < 0.05), but expression and relative synthesis of collagen I (normalized to cell proliferation) were significantly reduced.

PRP contained high amounts of IGF-1 and lower levels of TGF-β1 and PDGF-AB. The amounts of BMP-7 and -12 were below the detection limits.

**Conclusions:**

PRP is a source of growth factors such involved with tendon-bone healing. PRP had an anabolic effect on the human rotator cuff tenocytes of the same individual in vitro by means of cell proliferation and absolute, but not relative collagen I synthesis.

These results encourage further studies on clinical outcomes with more comparable standards in terms of preparation and application methods.

**Level of evidence:**

Controlled laboratory study.

## Background

The ongoing development of new rotator cuff (RC) repair techniques, such as double row repairs, has led to increased initial biomechanical stability, but recent meta-analyses have revealed that there is no clear clinical and radiographic advantage in terms of improved tendon integration [9, 15, 39, 42, 43, 48, 50]. As non-healing still represents the main challenge following surgical rotator cuff repair, biological approaches have been suggested as a way to improve tendon-bone healing [20, 23, 29, 36]. The use of growth factors seems to improve biological regeneration, but many of these are recombinant in origin and are often cost intensive to acquire [31].

Platelet rich plasma (PRP) as a supraphysiological concentration of platelets is an easily producible, low-cost source of several autologous growth factors [33]. However, available studies on the effect of PRP on clinical and radiographic parameters following RC repair do not just differ in their preparation and application methods, but also in their results. Recent meta-analyses have described either no clinical benefit or no consistent effect on re-tear rates, or at least uncertainty about the evidence [5, 7, 30, 47, 51, 58, 60].

In-vitro studies on the effect of PRP have, however, described improved tendon matrix production and gene expression, but only PRP preparations that are allogenic in origin were applied to tenocyte cultures, mainly with no dedicated characterization, and these were primarily obtained from monadic numbers of donors [12, 13, 17, 22, 24, 46]. In contrast, the process of assessing tendon cell cultures with autologous PRP from the same patient matches with the clinical application, but has not been described in the literature. This approach requires a protocol to first harvest and culture cells, followed by subsequent PRP production and application. Tendon cell characterization should also be part of the approach to verify the targeted cell type.

The presence of several growth factors involved with musculoskeletal repair, such as platelet-derived growth factor (PDGF), transforming growth factor-β (TGF-β), insulin-like growth factor (IGF), epidermal growth factor (EGF), vascular endothelial growth factor (VEGF), and fibroblast growth factor (FGF), has been described within PRP preparations [25, 33, 36, 52]. A focus on tenocyte metabolism and improved tendon-bone healing has highlighted further growth factors, such as bone morphogenetic protein 7 (BMP-7) [26, 27, 40, 56, 62] and 12 (BMP-12) [29, 49, 59]. However, it is currently unknown whether these are present in PRP.

The present study aims to characterize a human PRP-preparation in terms of the quantity of various growth factors. In addition, it investigates the effect of autologous PRP on tenocyte-like cells for the first time.

We hypothesize that there will be improved cell proliferation and collagen I synthesis under stimulation with PRP.

## Methods

### Study design, inclusion and exclusion criteria

After receiving institutional review board approval, supraspinatus (SSP) tendon samples were taken intraoperatively from *n* = 24 patients undergoing arthroscopic RC repair. Participation in the study did not affect the intra- and postoperative treatment strategy.

(*N* = 12) male and (*n* = 12) female patients with full thickness tears of the SSP tendon were included in the study. The exclusion criteria were partial- or massive tears (larger than Bateman III° [2]), tears with fatty infiltration greater than Goutallier III° [18], and medical conditions precluding arthroscopic cuff repair.

### Donor demographics, tendon material, surgery (*t* = 0)

*N* = 12 female (Ø age 64.5 years; range 50–74 years) and *n* = 12 male patients (Ø age 62.4 years; range, 38–73 years) were included in the study and subdivided into four groups by age (>/< 65 years) and sex. The average age of all the patients at the time of surgery was 63.5 years.

### Cell isolation and culture

During arthroscopic RC repair and after debridement with a shaver, all tendon biopsies were obtained 3-5 mm from the torn proximal tendon edge. Human tenocyte-like cells (hTLCs) were isolated from these SSP tendon biopsies by digestion with 0.3% collagenase type CLS II in PBS with Ca^2+^/Mg^2+^ for 2 h at 37 °C, as described previously [41]. The cells were cultured with a normal growth medium (DMEM/Ham’s F12 with 10% fetal calf serum (FCS), and 1% Penicillin/Streptomycin, all Biochrom AG, Berlin, Germany) at 37 °C, with 95% humidity and 5% carbon dioxide, and with a change of medium every 2 to 3 days. The cells were cryo-preserved until they were used for the stimulation when they reached a minimum of 5 × 10^5^ vital cells.

### Exemplary hTLC characterization

A total of 8 of the 24 hTLC cultures were characterized according to previously published research [41]. The cells were analyzed for their expression of the tendon markers collagen I, scleraxis, and Mohawk homeobox, and the negative markers collagen II and osteocalcin. The relative gene expression levels were normalized to glyceraldehyde 3-phosphate dehydrogenase (GAPDH) and calculated using the 2^-ΔCt^ method.

### PRP preparation

Approximately 2.5 years after their surgery, the 12 male and 12 female SSP tendon patients were invited to donate blood for the PRP preparation. The fresh PRP was then used for the autologous stimulation of the hTLCs, which had previously been isolated from their torn SSP biopsies and stored until used for stimulation experiments. A commercially available kit was used for the standardized PRP preparation according to the manufacturer’s instructions: Arthrex ACP® (Arthrex, Naples, FL), using 15 mL Arthrex ACP® double syringes. An average of 5 mL of PRP was obtained from each of them. No additional anticoagulants were used. Hence, quick preparation and processing of the PRP were mandatory.

Directly after preparation, the PRP was analyzed for the platelet count using a haemocytometer (C-Chip, NanoEnTek, Seoul, Korea). After preparation, the PRP was used to stimulate the hTLCs, and was then subsequently cryo-preserved at − 20 °C until the growth factor quantification.

### PRP-stimulation

**Fig. 1:**
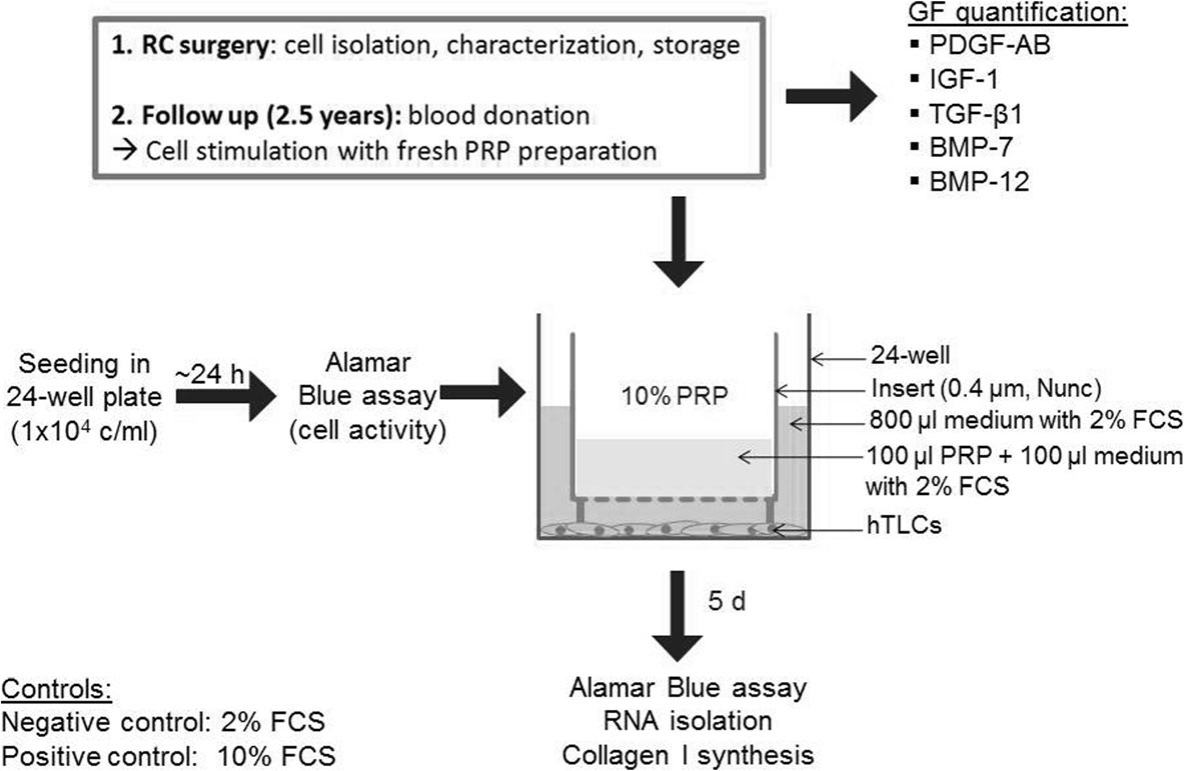
Synopsis of the experimental set up

A total of 1 × 10^4^ vital cells per well were seeded in a 24-well plate in triplicate and cultured in a normal growth medium. One day after seeding, an alamarBlue assay (Biozym, Germany) was used to analyze the cell proliferation. The cells then received either a growth medium with 2% FCS (negative control), a growth medium with 10% FCS (positive control), or 10% PRP in a growth medium with 2% FCS. Six wells were used to obtain enough RNA for the negative control. The PRP was applied to the cells in a polycarbonate cell culture insert with a pore size of 0.4 μm (Nunc), which was placed on the cell layer. The experimental set up is shown in Fig. 1. After incubating the cells for 5 days at 37 °C, the cell proliferation was analyzed, the cell culture supernatant was collected for the analysis of the collagen I protein synthesis, and the RNA was isolated to analyze the gene expression.

### Analysis of cell proliferation

An alamarBlue assay was used to indirectly measure cell proliferation by using the reducing power of viable cells. The medium was removed from the cells and 700 μl of a 10% alamarBlue solution in a medium with 10% FCS was applied to the cells and they were then incubated for 3 h at 37 °C. Subsequently, each well was analyzed in duplicate using spectrophotometry at 570 nm versus 595 nm against the blank (10% alamarBlue solution incubated without cells).

### Analysis of the collagen I protein synthesis

The collagen I protein synthesis was measured from day-5 cryo-preserved cell culture supernatants after PRP stimulation with the MicroVue C1CP EIA kit (TECOmedical, Germany). The collagen I protein synthesis was normalized to the cell proliferation on day 5.

### Analysis of gene expression

RNA was isolated from the cells on day 5 directly in the well using the NucleoSpin RNA II Kit (Machary Nagel, Germany) according to the manufacturer’s instructions. Cells in triplicate/6 repeats (negative control) from each donor were pooled. RNA quantity and purity were analyzed with a Nanodrop ND-1000 spectrophotometer (PeqLab Biotechnologie, Germany). A total of 100 ng of RNA was transcribed into complementary DNA (cDNA) using the qScript cDNA Supermix (Quanta BioSciences, USA) with the Epgradient Mastercycler (Eppendorf, Germany). For the quantitative real-time PCR (qRT-PCR) analysis, 1.25 ng of cDNA was used as the PCR template. A total of 20 μl of Sybr Green mastermix was used for each well and consisted of: 12.5 ml of Sybr Green Supermix (Quanta BioSciences), 6.5 μl of RNase/DNase-free water, and 1 μl of a primer mix (10 μM). All the primer sequences were designed using the Primer 3 software (Freeware; http://frodo.wi.mit.edu/primer3), and were produced by Tib Molbiol, Berlin, Germany (Table 1). A qRT-PCR program with an initial denaturation step for 3 min at 94 °C was used, followed by an amplification program with 40 repeated cycles (95 °C for 15 s, amplification temperature for 45 s, 72 °C for 30 s), and a melting curve program. The results were analyzed with the Realplex Software (Eppendorf). The relative gene expression levels were normalized to GAPDH and calculated using the 2^-ΔCt^ method.

**Table 1:** Primer

Target gene	Accession number	Product size [bp]	Primer sequence [5′ – 3′]
GAPDH	NM_002046	115	Forward: CCACTCCTCCACCTTTGACG Reverse: CATGAGGTCCACCACCCTGT
Col-I	NM_000088	197	Forward: TGACCTCAAGATGTGCCACT Reverse: ACCAGACATGCCTCTTGTCC
Col-III	NM_000090	199	Forward: GCTGGCATCAAAGGACATCG Reverse: TGTTACCTCGAGGCCCTGGT
Osteocalcin	NM_199173	209	Forward: CCCAGGCGCTACCTGTATCAA Reverse: CTGGAGAGGAGCAGAACTGG

### Growth factor quantification from the PRP

Growth factors such as PDGF-AB, IGF-I, TGF-β1, BMP-7, and BMP-12 (also known as growth and differentiation factor-7, GDF-7) were analyzed using ELISA according to the manufacturer’s instructions. The PRP was thawed and clotted for about 30 min at room temperature and centrifuged at 1000 × g for 10 min before being used for the ELISA analysis. The details for the ELISA kits are set out in Table 2. The growth factor concentration was normalized to the total protein content of the PRP, which was analyzed by a Coomassie Plus Protein Assay (Thermo Fisher Scientific, Germany).

**Table 2:** ELISA Kits

Analyte	ELISA Kit	Specifics	Dilution of PRP
PDGF-AB	Quantikine human PDGF-AB (R&D systems)		1:50
IGF-I	Quantikine human IGF-I (R&D systems)	PRP needs pre-treatment (solution provided in the kit)	1:100 (including pre-treatment)
TGF-β1	Quantikine human TGF-β1 (R&D systems)	PRP needs activation with 1 N HCL, neutralization with 1.2 N NaOH/0.5 M HEPES)	1:40 (including activation)
BMP-7	DuoSet human BMP-7 (R&D systems)		Undiluted
BMP-12 (GDF-7)	Human GDF-7 ELISA Kit (Blue Gene)	Competitive ELISA	1:2

### Statistics

A statistical analysis was performed for 4 individual groups (*n* = 6) using the Mann-Whitney-U Test. The *P*-value was set to *p* ≤ 0.05 and adjusted using the Bonferroni Holm correction. A Spearman’s Rho correlation (r_s_) was used to analyze the correlation of different parameters.

## Results

### Exemplary hTLC characterization

The exemplary characterization of 8 hTLC cultures revealed high amounts of collagen I (2^-ΔCt^ = 2.38 ± 0.85) and an abundance of the tendon markers scleraxis (2^-ΔCt^ = 0.003 ± 0.002) and Mohawk homeobox (2^-ΔCt^ = 0.015 ± 0.006) in accordance with previous studies [41]. Osteocalcin expression was weak in all the cells (2^-ΔCt^ = 0.0006 ± 0.0002), while collagen II was only present in 5 of the 8 hTLC cultures at a negligible level (2^-ΔCt^ = 0.00001 ± 0.00001).

### Platelet and growth factor quantification in the PRP

**Fig. 2:**
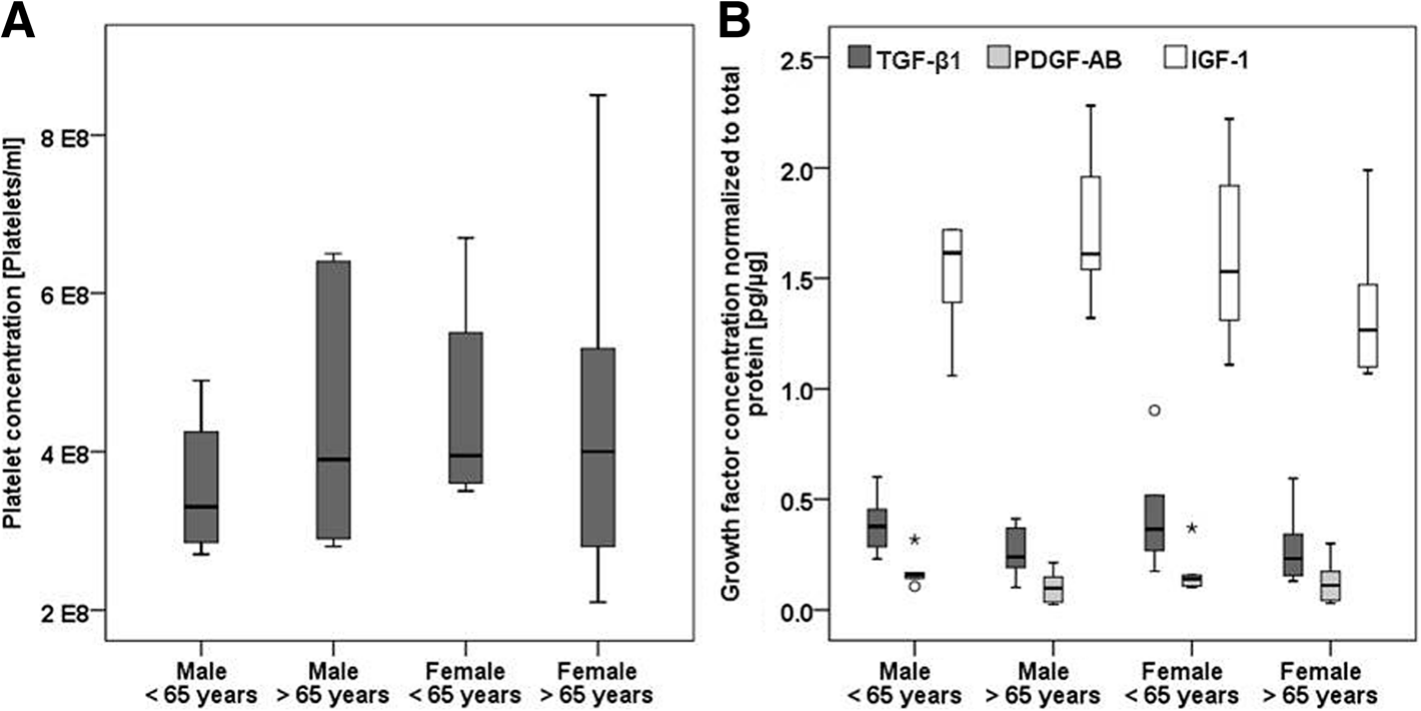
**a** Platelet and **b** growth factor concentrations in the PRP of the four investigated donor groups. No significant differences were found between the groups. Stars and circles mark the outliers

The platelet concentrations showed no significant differences between the 4 investigated donor groups. The median of the platelets was 3.6 × 10^8^ platelets/ml for the younger male group and 4.5 × 10^8^ platelets/ml for the 3 other groups (Fig. 2a).

The growth factor quantification revealed no significant differences between the groups. IGF-1 had the highest concentrations in the PRP, followed by TGF-β1 and PDGF-AB (Fig. 2b). Only 1 of the 24 PRPs contained a very low amount of BMP-7 (0.0004 pg/μg), while low levels of BMP-12 (0.021 pg/μg) were detected in 1 other sample. The PDGF-AB concentrations significantly correlated with the platelet concentrations in the PRP (r_s_ = 0.469, *p* = 0.037). The correlation between the TGF-β1 and PDGF-AB concentrations was also significant (r_s_ = 0.877, *p* < 0.001).

### Cell proliferation

**Fig. 3:**
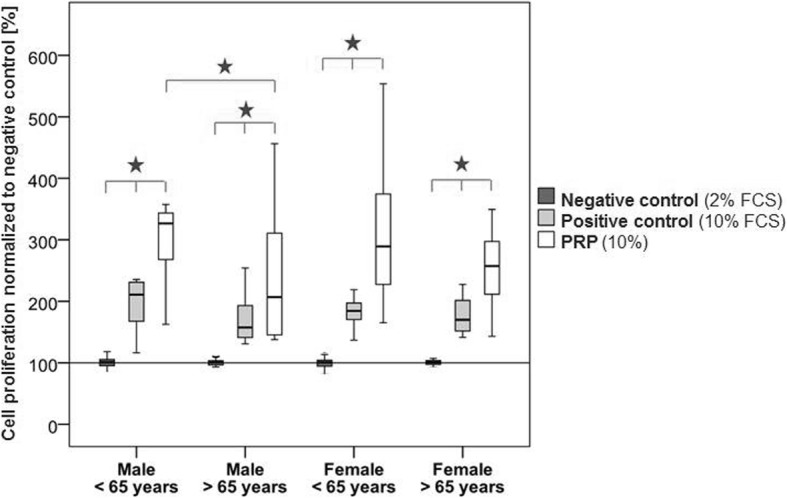
Cell proliferation after PRP stimulation in the four investigated donor groups normalized to the negative control. The PRP led to significantly increased cell proliferation compared to the positive and negative controls. Additionally, the positive control showed an elevated cell proliferation compared to the negative control. Cell proliferation in the PRP-treated cells was significantly reduced in the older compared to the younger male group Stars indicate significant differences between the groups (*p* ≤ 0.05)

Cell proliferation was significantly increased in all the groups by stimulation with PRP compared to the positive (10% FCS, *p* ≤ 0.048) and negative controls (2% FCS, *p* ≤ 0.0001). Significantly increased cell proliferation was further observed for all the hTLCs of the positive compared to the negative control group (p ≤ 0.0001). Additionally, in the group with the males over 65 years of age, the PRP showed significantly reduced stimulation potential compared to the group with the males under 65 years of age (*p* = 0.009). The cell proliferation did not correlate with the platelet or the growth factor concentrations of the PRP (Fig. 3).

### Collagen I protein synthesis

**Fig. 4:**
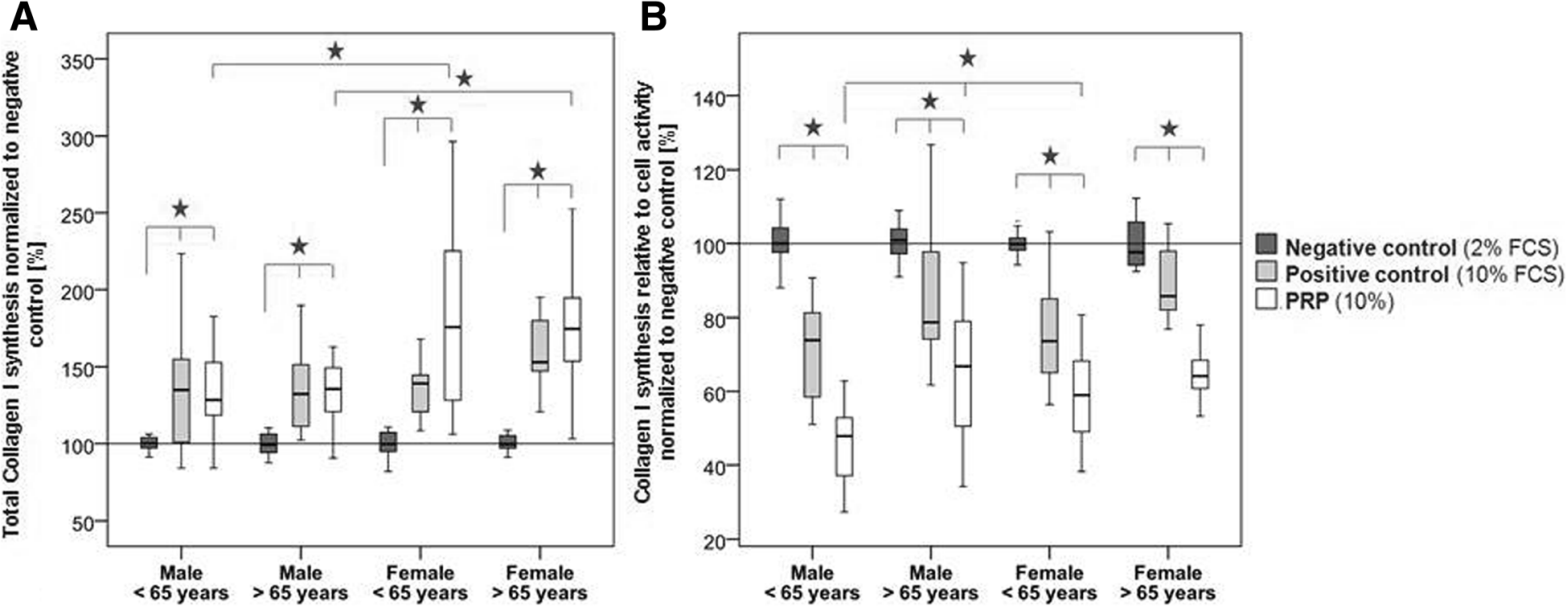
Total and relative collagen I synthesis normalized to the negative control. **a** The total collagen I synthesis was significantly increased in the PRP group and the positive control compared to the negative control. PRP treatment led to significantly elevated effects in the hTLCs of the older compared to the younger donors. **b** Collagen I synthesis relative to cell proliferation was significantly reduced in the PRP group compared to the positive and negative controls, as well as in the positive compared to the negative control. In the younger male donors, the relative collagen I synthesis was significantly reduced compared to the older male and younger female groups. Stars indicate significant differences at p ≤ 0.05 between groups

In all the analyzed groups, the absolute collagen I synthesis (without normalization to cell proliferation) was significantly increased in the PRP-stimulated cells and the positive control group (10% FCS) compared to the negative control group (2% FCS, *p* ≤ 0.005), but not between the positive control (10% FCS) and the PRP-treated cells. In addition, the PRP stimulation of the female donors (>/< 65 years) resulted in a significantly higher increase in total collagen I synthesis compared to the respective male groups (< 65 years: *p* = 0.043, > 65 years: *p* = 0.002) (Fig. 4a).

With normalization to cell proliferation, the relative collagen I synthesis was significantly reduced in the PRP-treated cells compared to the hTLCs of the positive (10% FCS, *p* ≤ 0.01) and negative controls (2% FCS, *p* ≤ 0.0001), as well as in the positive compared to the negative control (*p* ≤ 0.03). In the younger male group, the PRP stimulation led to significantly reduced collagen I synthesis compared to the older male (*p* = 0.003) and the younger female groups (*p* = 0.006) (Fig. 4b). Correlation analysis revealed a significant negative correlation between the PDGF-AB concentration and the relative collagen I synthesis (r_s_ = − 0.502, *p* = 0.017).

### Gene expression

**Fig. 5:**
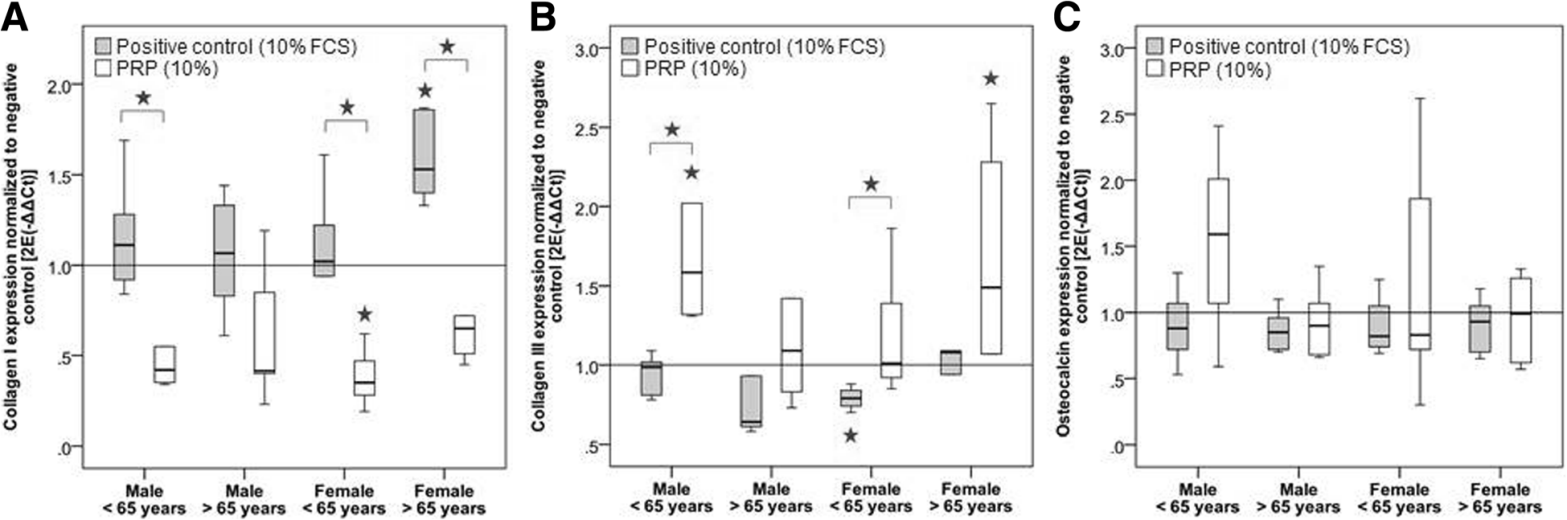
Relative gene expression measured by qRT-PCR and normalized to GAPDH and the negative control (line). Stars above brackets mark significant differences between these groups at *p* ≤ 0.05. Stars alone mark significant differences to the negative control. **a** Collagen I expression was significantly reduced in all the investigated groups except the older male group compared to the positive control. **b** The expression of collagen III was significantly increased in the PRP-treated cells compared to the positive and/or negative control in all the groups except the older males. **c** No significant differences were demonstrated for the osteocalcin expression

Collagen I expression was significantly reduced after PRP treatment compared to the positive control in the younger male (*p* = 0.016) and older female (p = 0.016) groups. Additionally, in the hTLCs of the female donors over the age of 65, the collagen I expression in the positive control was significantly increased compared to the negative control (*p* = 0.005). In the younger female group, the collagen I expression was significantly reduced compared to the negative (p = 0.005) and positive controls (*p* = 0.009). No significant differences were detected in the older male group (Fig. 5a).

The collagen III expression showed significant elevation in the male group under the age of 65 compared to the negative (*p* = 0.002) and positive controls (*p* = 0.004). In the hTLCs of the female donors under 65, the collagen III expression was induced in the PRP-treated cells compared to the positive control (*p* = 0.016), but it was significantly lower in the positive compared to the negative control (*p* = 0.005). In the cells of the older female donors, the collagen III expression was significantly increased in the PRP compared to the negative control (*p* = 0.007). No significant differences were observed for the older male group (Fig. 5b).

The osteocalcin expression was unaffected by the PRP or FCS treatment of the hTLCs (Fig. 5c).

No correlations were detected between the growth factor content in the PRP and the gene expression of the hTLCs.

## Discussion

The current study for the first time describes the stimulating effects of autologous PRP on the same patients tenocytes of the human rotator cuff. We hereby assessed parameters such as tenocyte proliferation as well as expression and synthesis of extracellular matrix components, particularly collagen I and III. were significantly improved (without normalization to cell proliferation). As discussed later, this finding is in general accordance with other in vitro studies in the field that describe enhanced cell proliferation, tendon matrix synthesis, and gene expression under allogenic PRP of different configurations [13, 17, 22, 24, 46].

The effect of PRP was investigated in hTLCs of female and male subgroups of patients who were under and above 65 years of age. Some clinical cohort studies had suggested that a higher patient age is related to unsuccessful RC repair, but no consistent age limits have been defined [3, 11, 14, 19, 35, 37, 53, 61]. Furthermore, a *sex*-related effect had been advocated by some authors [10, 19], while others could not confirm this observation [35, 38, 54, 61].

If patient age or sex was a critical issue, standard RC repairs would be unsatisfactory in those beyond certain limits, possibly demanding additional biological RC repair augmentation, for example with growth factors obtained with PRP [1, 23, 24, 31, 40, 45, 49].

If, for instance, elderly female patients required additional biologic augmentation, autologous PRP might be one possible approach, as suggested in the present study. Recent in vitro studies have described age-related differences in biologic RC cell potential among male and female patients, with a weaker potential in those above the age of 65 [27, 28]. Tenocyte-like cells can be stimulated with growth factors such as BMP-2 and -7 by means of increased cell activity and collagen I synthesis in elderly male and female patients [27, 28]. As discussed later, BMP-7 could not be quantified in the PRP preparation approach currently employed. However, the blend of other autologous growth factors herein demonstrated stimulating effects, suggesting that PRP might have potential when it comes to augmentation, particularly in this RC repair patient cohort with a possible biological demand.

Several methodological features of the present research differ from existing in vitro studies on PRP and tendon cells and provide new insights. A major strength of this study is that it is the first approach to investigate the effect of PRP on supraspinatus tenocytes of the same patient, representing the clinical and intraoperative application of autologous PRP. This approach requires a more elaborate protocol to allow for intraoperative tendon harvest, subsequent cell isolation, and timed cell cultivation prior to scheduling patients for blood samples, PRP preparation, and application.

Furthermore, the present study features the largest number of enrolled donor patients of either sex or age group, and thus represents the typical patient cohort undergoing RC repair. Other studies in the field have assessed only monadic numbers of donors with allogenic PRP preparations [13, 17, 22, 24, 46]. Cross et al. involved *n* = 20 RC samples, but these received allogenic PRP preparations and had irreparable and long-standing RC tears (cuff tear arthropathy), which does not represent the patient cohort undergoing RC repair (in contrast to the donors in the present study) [12].

Another strength of this study is that tissue samples were obtained from torn rotator cuffs at the site of repair (in contrast to intact rotator cuff material [55]). This may provide more specific insight into the biological effect of PRP on the targeted type of rotator cuff tissues when compared to tendon cultures with other origins [12]. In this regard, de Mos et al. suggested that tenocytes from degenerated and torn RCs might respond differently to PRP than the intact hamstring tendon cells of the young individuals investigated in their study [13]. However, their observation of increased tenocyte proliferation is in accordance with our present findings. The authors furthermore suggested a possible decrease of collagen synthesis per cell which is confirmed in our results, where total collagen I protein synthesis was increased but relative synthesis (normalized to cell proliferation) was significantly reduced. Could this be one reason why current clinical studies with this regard are rather disencouraging? With respect to the increased cell proliferation we believe it is more precise to differentiate between total and relative collagen I synthesis, even though it remains a gap of knowledge which of these parameters is the most relevant for successful clinical transfer. In addition, de Mos et al. observed reduced gene expression of collagen I, which again is in accordance with the present findings in torn RC tenocytes. The authors also described upregulation of matrix-degrading enzymes (matrix metalloproteinase – MMP - 1 and 3) in PRP-exposed cells, and concluded that PRP might accelerate the catabolic demarcation of traumatically injured tendon matrices [13]. We cannot compare these results to degenerative, torn RC tissue, as MMP expression was not assessed in the present study. However, Cross et al. described reduced matrix degradation and inflammation cytokines in elderly, torn RC-tenocytes treated with the same leukocyte-poor PRP preparation set as in the present study (Arthrex ACP®).

In the current research, tendon cell characterization was conducted prior to studying the cell response. This should be considered important enough to exclude the analysis of unspecific fibroblast cultures [41], and was conducted by some authors assessing the effect of PRP on tendon cells after characterization for tendon-specific markers such as scleraxis or decorin [17, 24]. In rodent tendon stem cells, PRP induced tenocyte differentiation, while adipocyte, chondrocyte, and osteocyte lineages were suppressed [8]. This reflects the present findings in human tenocytes, namely that osteocalcin expression was unaffected by PRP application.

A weakness of the present study is that no different PRP dosages were assessed, as other authors in this field have described previously [13, 17, 46]. The dose-dependent response of tenocytes to applied growth factors has, however, been reported elsewhere [40, 62].

Growth factors (such as BMP 2 and 7) have been found to stimulate human RC cells, but dosages beyond the ideal concentration reduced tendon cell proliferation and collagen production [40]. Sadoghi et al. found a significant effect of PRP on RC fibroblast proliferation in vitro, with an optimal benefit using a onefold or 5-fold, but not 10-fold, PRP concentration [46]. Further studies have demonstrated that increasing platelet concentrations in PRP applications beyond an ideal level does not result in further anabolic upregulation (in animal/human tenocytes) [4, 17]*.* In contrast, (too) high concentrations have been shown to have inhibitory effects on proliferation and collagen production, while MMP production increased, which is possibly counterproductive when it comes to tendon bone regeneration [4, 17]. It has been suggested that an over-stimulation of cells might result in adverse effects, such as poorly-differentiated scar tissue at the insertion site [6, 57]. Accordingly, the chosen PRP concentration in the present study may not have been optimal, as the relative collagen I synthesis was significantly reduced (when normalized to cell proliferation). Moreover, an increase in PDGF-AB concentration in the PRP was found to correlate with a reduced relative collagen I synthesis, indicating that lower PRP concentrations could be beneficial. However, even if the optimal PRP dosage in vitro is known, it nevertheless remains challenging to transfer this knowledge to the clinical setting. It is technically difficult to influence or optimize growth factor dosages in the final PRP product intraoperatively, as this correlates to plasma viscosity and concentration at the time of venous blood sampling.

As the stimulation of osteoblasts is helpful for re-establishing the tendon-bone footprint following RC repair, growth factors with bone and tendon-stimulating potential (such as BMP-7 [26, 27, 40, 56, 62] and − 12 [29, 49, 59]) might be beneficial at the repair site. Accordingly, the current study aimed to quantify the amount of these factors to gain further insight, but the quantities were below the technical detection limit except for 1 donor with a low but detectable level of BMP-7 and 1 donor with a low level of BMP-12. This could either be a technical issue related to insufficient sensitivity of the kits or the fact that these respective growth factors are indeed absent in the PRP preparations.

To date, more clinical and radiographic studies on PRP and RC repairs have been published than in vitro studies on the biological characteristics of available PRP products. Some studies suggested a pain reduction following PRP application [16, 44]. However, recent reviews and meta-analyses found no significant effects of PRP augmentation in the treatment of full thickness RC tear repairs by means of outcome scores or re-tear rates [5, 7, 21, 30, 47, 51, 60, 63].

The question arises as to why the majority of clinical and radiographic studies on PRP augmentation in RC repair do not confirm the promising in vitro studies that generally describe positive effects of PRP on RC tendon cells.

One issue is the growth factor dose-dependent response of tendon cells and the difficulty of affecting their concentration in clinical practice, as discussed above. Yet other factors may also account for this controversy. Any analysis of the literature is complicated by the lack of standardization of study protocols, PRP preparation techniques, and the respective outcome measures in clinical studies [51].

There is a broad range of PRP preparations distributed in the market and employed in several studies. Yet it is not just the content and concentrations of platelets, white blood cells, and growth factors that differ significantly between different products and methods (1- versus double spin process), but also intra-individual variations of PRP products [12, 32, 34]. This variation may contribute to inhomogenous results, as may further variables in clinical studies such as the application time (intraoperatively/days later) and site (underneath the reconstructed tendon-bone unit or over the top) [60]. Finally, the actual surgical reconstruction technique (single vs. double row repair) and postoperative rehab protocols may further account for differences and hinder comparability.

These variables might contribute to the understanding of heterogeneous results in clinical and radiographic studies and the demand for further translational studies, while PRP generally shows effects on human RC tendon cells in vitro.

## Conclusions

In this study, autologous PRP was a source of growth factors such as IGF-1, TGF-β, and PDGF-AB, but no detectable amounts of BMP-7 and -12 were found. PRP had an anabolic effect on the human rotator cuff tenocytes of the same individual in vitro by means of increased cell proliferation. On the other hand, and absolute, but nor relative collagen I synthesis and gene expression was significantly reduced and might explain limited clinical results of PRP applications. These results encourage further studies on clinical outcomes with more comparable standards in terms of preparation and application methods.

## Abbreviations

BMP: Bone morphogenetic protein
COL: Collagen
hTLC: Human tenocyte-like cells
IGF-1: Insulin-like growth factor
PDGF: Platelet-derived growth factor
PRP: Platelet rich plasma
RC: Rotator cuff
SSP: Supraspinatus
TGF-β: Transforming growth factor
